# Influence of Infectious Pancreatic Necrosis Virus and *Yersinia ruckeri* Co-Infection on a Non-Specific Immune System in Rainbow Trout (*Oncorhynchus mykiss*)

**DOI:** 10.3390/ani11071974

**Published:** 2021-07-02

**Authors:** Joanna Pajdak-Czaus, Patrycja Schulz, Elżbieta Terech-Majewska, Wojciech Szweda, Andrzej Krzysztof Siwicki, Aleksandra Platt-Samoraj

**Affiliations:** 1 Department of Epizootiology, Faculty of Veterinary Medicine, University of Warmia and Mazury in Olsztyn, Oczapowskiego 13, 10-718 Olsztyn, Poland; etam@uwm.edu.pl (E.T.-M.); szweda@uwm.edu.pl (W.S.); platt@uwm.edu.pl (A.P.-S.); 2 Department of Ichthyopathology and Fish Health Prevention, S. Sakowicz Inland Fisheries Institute, Główna 48, 05-500 Żabieniec, Poland; p.schulz@infish.com.pl; 3 Department of Microbiology and Clinical Immunology, Faculty of Veterinary Medicine, University of Warmia and Mazury in Olsztyn, Oczapowskiego 13, 10-719 Olsztyn, Poland; siwicki@uwm.edu.pl

**Keywords:** IPNV, enteric redmouth disease, mixed infection, fish pathogens, aquaculture

## Abstract

**Simple Summary:**

Although the intensification of fish production allows for better economic results, it also increases the risk of infections depending on fish density. Frequently occurring co-infections are difficult to diagnose because the isolated microorganisms are opportunistic, and their role in the development of disease is uncertain. The infectious pancreatic necrosis virus (IPNV) and bacteria *Yersinia ruckeri* are widespread pathogens of rainbow trout, causing economic losses in fish culture. The influence of the studied pathogens on non-specific immunity in both single and co-infections was determined. Results imply that IPNV infection may contribute to secondary bacterial infections.

**Abstract:**

Background: The IPNV is one of the most common viral pathogens of rainbow trout (*Oncorhynchus mykiss*), while *Y. ruckeri* infections are widespread among bacterial agents. The current study aimed to determine the influence of IPNV and *Y. ruckeri* co-infection on a non-specific immune response. Methods: Two experiments were conducted. The first experiment determined the changes in non-specific immunity parameters upon the simultaneous occurrence of IPNV and *Y. ruckeri* infection. In the second experiment, infection with the IPNV was performed two weeks before *Y. ruckeri* infection. The level of total protein, gamma globulins, the activity of lysozyme and ceruloplasmin, as well as the metabolic activity and potential killing activity of phagocytes were measured: 0, 24 h, 72 h, 7 days, 14 days, and 21 days after co-infection. Results: A differentiated effect on the parameters of the non-specific immune response was shown between single infections with the IPNV and *Y. ruckeri* as well as co-infection with these pathogens. Conclusions: The immune response in the course of a co-infection depended on the time between infections. IPNV infection causes lysozyme activity suppression, which may lead to secondary bacterial infections.

## 1. Introduction

Due to the growing human population, the demand for wholesome food products, including fish, is increasing and aquaculture has become one of the fastest-growing sectors of food production. As a food product, fish are a rich source of protein, fatty acids, vitamins and micro- and macro-elements. Based on statistics, it is estimated that the global per capita consumption of fish has doubled since 1960. Fish production should increase by 18% by the year 2022 to meet the future needs of the growing human population [1]. One fast-growing sector is rainbow trout (*Oncorhynchus mykiss*) farming.

In 2000, world production of rainbow trout amounted to almost 496,000 tons, and in 2016 it increased to 814,000 tons [2]. Limitations in the development of this species’ production include its high requirements for water quality, particularly oxygen levels. Infectious and parasitic diseases are another challenge for breeders [3]. Many of the microorganisms are opportunistic and cause the development of clinical symptoms in certain situations. The predisposing factors include changes in the physicochemical parameters of water, breeding procedures such as fish sorting, and excessive density in tanks. This is particularly important because the overcrowding of fish causes stress that can lead to disease in asymptomatic vectors, and a large group of fish promotes the spread of pathogens. Among the most important diseases of salmonids are infectious pancreatic necrosis (IPN) and enteric redmouth disease (ERM).

IPN is a viral disease caused by the infectious pancreatic necrosis virus (IPNV) belonging to the *Birnaviridae* family. Salmonids are the most susceptible to the disease, but the IPNV has also been isolated from affected individuals of other fish species. In the case of horizontal transmission, the virus penetrates the gills, the intestinal epithelium, and certain parts of the skin. After a few days, the virus is detectable in leukocytes and is distributed through the bloodstream to most tissues, including the kidneys, spleen, pancreas, liver, heart, brain, skin, intestine, and reproductive cells. Cell tropism depends on the host species, virus strain, and age of the fish. In fry, the virus replicates intensively in most tissues and causes necrosis of the pancreas and intestinal mucosa. The virus can “hide” and dwell inside leukocytes. This is one of the possible causes of vaccination not being effective. 

It has been proven that the level of vaccine immunity is not sufficient to protect against the disease, and in stressful situations, fish may develop clinical symptoms [4,5]. The highest mortality rate (up to 100%) is recorded in fry and the post-smolt stage when the fish are transferred to sea cages. In older fish, infection is usually asymptomatic. The survivors of outbreaks become carriers of the virus and can be a source of infection for other individuals. The carrier status may last for several years. Due to its high stability under various conditions, water is a frequent route of transmission. Most disease symptoms are not specific and common to most viral fish diseases. The first alarming symptom is the increase in mortality, which is sudden and increases over time. Symptoms include exophthalmos, darkening of the skin, pallor of the gills, a distended abdomen, the presence of a mucous pseudo-cast, and skin ecchymoses. Sick fish are lethargic and lose their appetite. The only characteristic symptom is corkscrew movements while swimming. Autopsy lesions include pallor of the liver and spleen, empty gastrointestinal tract, and spot ecchymoses, especially in the peripancreatic tissue between pyloric caeca [3,4]. The virus spreads not only horizontally but also vertically. It can be transported both on the surface and inside the spawn. For this reason, spawners should be rigorously selected, and only IPNV-free individuals should be intended for breeding [3,5].

*Yersinia ruckeri* (*Y. ruckeri*) is a bacterium with a global distribution. It is an etiological factor of ERM. The name of the disease comes from a characteristic symptom in the form of petechiae around the mouth [6]. Rainbow trout are considered the most susceptible to the disease, however *Y. ruckeri* was also isolated from other fish species, both from clinically healthy fish as well as from infected individuals [7]. The development of an asymptomatic carrier state after disease outbreaks may affect up to 25% of clinically healthy trout. The survival of *Y. ruckeri* in the aquatic environment may persist for more than three months [8]. Strains showing movement can form a biofilm [9,10]. These abilities are the cause of the recurring, cyclical occurrence of the disease in some farms. Water invertebrates and birds also play a role in spreading the disease [11]. The infection route is most likely through the gill epithelium [6,12]. It is then distributed through the bloodstream to all internal organs, including the brain [13]. ERM can occur in fish at any age but, most often, it affects fry up to the age of one. In this age group, the disease is often acute and, over time, may contribute to cumulative mortality of 30–35%. In addition to the characteristic symptom of a “red mouth”, petechiae can also be found inside the mouth on the tongue and palate and in the eyeball. Exophthalmos and darkening of the skin are also common symptoms. Pathological studies reveal splenomegaly, inflammation of the posterior intestine, which is often filled with thick yellow mucus and petechiae on serosa and in muscles [6,12,14].

Many literature reports have described the main pathogens of rainbow trout. Most of them concentrate on infections with one microorganism. In natural conditions, however, mixed infections (co-infections) occur more frequently. Co-infection is a situation in which an organism is infected with at least two genetically distinct microorganisms, and each of them has a damaging effect on the host organism. Interactions between pathogens can be synergistic or antagonistic. The synergistic effect occurs in particular when the first infectious agent causes immunosuppression, thus supporting the development of infection with other pathogens. The antagonistic mechanism most often occurs when microorganisms compete for receptor sites and substances needed for growth. For this reason, antagonism occurs mainly in infections with homologous pathogens, e.g., bacterial–bacterial or viral–viral. The relationship in bacterial–viral co-infections is usually synergistic [15]. There is information in the literature about the co-existence of the IPNV with other viruses. However, little is known about the interaction of the IPNV with bacterial pathogens.

The aim of the current study was to evaluate IPNV infection on the course of ERM, the influence of infection on a non-specific immune response, and an attempt to determine the relationship between IPNV and *Y. ruckeri* in co-infection.

## 2. Materials and Methods

The experiments were carried out in conformity with animal protection law and the recommendations of the Animal Ethics Committee of the University of Warmia and Mazury in Olsztyn No. 45/2016 and No. 40/2019.

### 2.1. Fish

Rainbow trout fry with an average body weight of 60–80 g were brought from a fishing farm with known epizootic status concerning the occurrence of IPNV and *Y. ruckeri*. 

After the fish were transported to the Fish Diseases Laboratory of the Department of Epizootiology, Faculty of Veterinary Medicine, University of Warmia and Mazury in Olsztyn, Poland, they underwent a two-week acclimatization. Samples were taken to confirm that the fish were free of the IPNV and *Y. ruckeri*. A PCR examination and (in the case of IPNV) a cell culture were conducted. The fish were kept in tanks with a capacity of 300 L supplied with tap water. Separate equipment was assigned to each tank to minimize the risk of transmitting pathogens. The temperature, the dissolved oxygen level and the pH were controlled during the experiment (SL 1000, Hach Lange, Wrocław, Poland). The fish were fed commercial feed using timer feeders in an amount appropriate to body weight and water temperature. The light intensity was regulated with a 12 × 12 h light cycle. Physicochemical conditions were maintained at the optimal levels: temperature 14–15 °C, O_2_- min 8 mg/L, pH 6.5–7.5.

### 2.2. Experimental Infection

Two experiments were conducted. Each experiment was performed in two parallel replications. After acclimatization, the fish were randomly divided into four experimental groups for both experiments. Each group contained 60 fish:

- group C—uninfected control;
- group Yer—infected with *Y. ruckeri*;
- group IPN—infected with IPNV;
- group Yer+IPN—co-infected with *Y. ruckeri* and IPNV.

#### 2.2.1. Experiment I

In the first experiment, the experimental group Yer was infected intraperitoneally with the pathogenic strain of *Y. ruckeri*. A strain isolated from the field case was used. It belonged to the O1 serotype and biotype 2. The infectious dose was 3 × 10 ^5^ CFU/g b.w. [16]. Group IPN was infected with the reference strain IPNV Sp at the dose of 10^4^ TCID_50_/g b.w. [17]. Group Yer+IPN was infected with both pathogens using the above-mentioned doses. Group C was the control group and received an intraperitoneal injection of sterile PBS.

#### 2.2.2. Experiment II

In the second experiment, groups IPN and Yer+IPN were infected first with the above-mentioned dose of IPNV. Groups C and Yer were injected with a clean virus transport medium. After two weeks and confirmation of the effectiveness of the infection, groups Yer and Yer+IPN were infected with *Y. ruckeri* with the above-mentioned dose. Groups C and IPN received an injection of sterile PBS.

During both experiments, the fish were observed twice a day for disease symptoms.

#### 2.2.3. Sampling

Five fish from each group were sampled on (counting from the day of infection with *Y. ruckeri*): day 0 (before infection), 24 h post-infection (p.i.), 72 h p.i., 7 days p.i., 14 days p.i., and 21 days p.i. Clinical and pathological examinations were performed to determine the presence of clinical symptoms of the disease. For immunological assays, spleen and blood serum were collected. Samples were taken to confirm the presence of examined pathogens in infected groups and the absence in the control group. IPN was diagnosed with the use of RT-PCR as well as a cell culture. *Y. ruckeri* was isolated on bacteriological mediums and identified via PCR [18].

### 2.3. Immunological Tests

The following parameters were determined: total protein level, gamma globulin level, lysozyme and ceruloplasmin activity, and metabolic and killing activity of phagocytes.

#### 2.3.1. Tests Performed on Blood Serum

Whole blood was centrifuged for 10 min at 8,500 rpm and 4 °C. Blood serum was collected and stored at −20 °C until further analysis. It was used to perform the following assays:

##### Total Protein

The total protein level was determined via the spectrophotometric method [19] using the Diagnostic Kits—Protein Total Reagents (Sigma-Aldrich, Taufkirchen, Germany).

##### Gamma Globulin Level

One tenth of a milliliter of serum was applied to a 96-well plate and 0.1 mL of 12% polyethylene glycol (10,000 kD) (Sigma-Aldrich) diluted with deionized water was added. The plates were incubated at room temperature for 2 h in a shaking incubator (Stuart, Orbital Incubator). The plates were then centrifuged for 10 min at 5000 rpm to separate the polyethylene glycol bound γ-globulin fraction from the remaining total protein (supernatant). The optical density (OD) was then measured at 620 nm. The OD of the supernatant was subtracted from the OD of the total protein [19].

##### Lysozyme Activity

Serum lysozyme activity was measured using a turbidimetric test carried out according to Siwicki and Anderson [19]. The test was based on the lysis of the lysozyme sensitive Gram-positive bacterium *Micrococcus lysodeikticus* (Sigma-Aldrich). The serum was diluted 1:1 with a sodium phosphate buffer, then 0.1 mL was applied to a microplate. Five tenths of a milliliter of *Micrococcus lysodeikticus* suspension (325 mg of bacteria/100 mL of phosphate buffer) was added. The plate was incubated at 25 °C. The absorbance was measured immediately after adding the bacteria and after 15 min of incubation at 450 nm. Egg white lysozyme (Sigma-Aldrich) was the standard. Mean OD values were calculated [19].

##### Ceruloplasmin Activity

The serum ceruloplasmin activity was determined according to the method described by Siwicki and Anderson [19]. The following buffers were prepared:

- acetate buffer (pH 5.2 containing crystalline acetic acid, sodium acetate trihydrate, and 15 mg EDTA);
- buffered substrate solution (0.2% p-phenylenediamine (PPD) in acetic buffer);
- sodium azide solution (0.02% sodium azide solution in deionized water).

In the next step, 0.5 mL of the buffered solution was transferred to each of two test tubes (16 × 100 mm) immersed in a water bath at 37 °C. One of the test tubes was the experimental sample, and the other was the control sample. Fifty microliters of serum was added to the experimental test tube, followed by incubation for 15 min. Following this, 2 mL of sodium azide solution was added to both test tubes. Fifty microliters of serum was added to the control tube, and both tubes were mixed. The OD of the test sample was read at 540 nm using the control sample as a reference, and the mean OD values were then calculated.

#### 2.3.2. Tests Performed on Tissue

Leukocytes for the tests were isolated from the fish spleen. Organs were aseptically removed from the fish and homogenized with the addition of 3 mL of Roswell Park Memorial Institute (RPMI)-1640 solution (Gibco) with an antibiotic (penicillin + streptomycin), 1 mL of antibiotic per 100 mL of RPMI. The suspension was transferred to test tubes and centrifuged for 15 min at 2500 rpm. The supernatant was decanted, and 4 mL of RPMI-1640 with antibiotic was added to the precipitate. New tubes with 3 mL of Gradisol L (Aqua-Medica, Łódź, Poland) were prepared, and the resulting suspension was layered on top of it. Tubes were centrifuged for 45 min at 4 °C and 2500 rpm. The resulting interphase was transferred to new tubes, and the solution of RPMI-1640 with antibiotic was added in a 1:1 ratio. The samples were centrifuged for 15 min at 4 °C and 2000 rpm. The supernatant was then decanted, and the RPMI-1640 solution containing 10% fetal bovine serum (FBS, Sigma-Aldrich) and a 1% mixture of antibiotic and fungicide (Sigma-Aldrich) solution was added to the pellet to obtain a concentration of 2 × 10 ^5^ cells/mL [20].

The metabolic activity of spleen phagocytes was measured by determining the size of the intracellular respiratory burst activity (RBA) after phorbol myristate acetate (PMA, Sigma-Aldrich) stimulation. The phagocyte-killing activity (PKA) was determined spectrophotometrically after stimulation with *Aeromonas* (*A.*) *hydrophila* [19]. RPMI-1640 with antibiotic (penicillin + streptomycin) and serum was applied to a 96-well plate, and then 100 µL of a previously prepared suspension of leukocytes isolated from the spleen was added. The plate was incubated for 24 h at 24 °C. After incubation, the following were applied:

- nitro blue tetrazolium (NBT)—0.1% NBT (Sigma-Aldrich) to the first two rows (two replicates);
- RBA—PMA solution with 0.1% NBT to the next three rows (three replicates);
- PKA—bacterial suspension and 0.1% NBT to the last three rows (triplicate).

The plates were incubated for 30 min at 24 °C, the fluid was decanted and washed twice with ethanol. Afterward, 2M KOH and dimethylsulfoxide (DMSO, Sigma-Aldrich) were added, and the amount of reduced NBT was read spectrophotometrically. The OD of the solution was measured colorimetrically on a Sunrise absorbance reader (Tecan Männedorf, Switzerland) at 620 nm. Data are expressed as mean values from replicate. 

### 2.4. Statistical Analysis

The statistical analysis was performed using the Statistica 13 program. The compliance of the empirical distribution with the normal distribution was tested using the Shapiro–Wilk test. The equality of variance was determined using Levene’s test. When the distribution was consistent with the normal distribution and the variance was equal, the obtained results were subjected to the analysis of variance (ANOVA) with repeated measurements. When the normality of the distribution was not shown and/or the variances were not equal, a non-parametric analysis of variance (Friedman ANOVA) was used. Statistically significant results were estimated using the Tukey’s or Dunn’s test (with non-parametric analysis), assuming a significance level of *p* < 0.05.

## 3. Results

### 3.1. Experiment I

**Figure 1:**
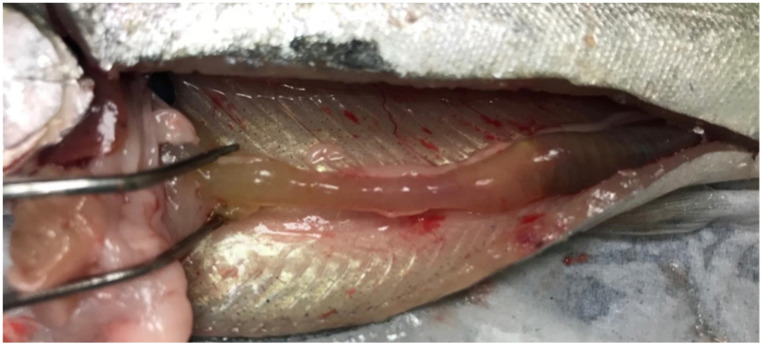
The digestive tract filled with yellow liquid content in the course of ERM.

**Figure 2:**
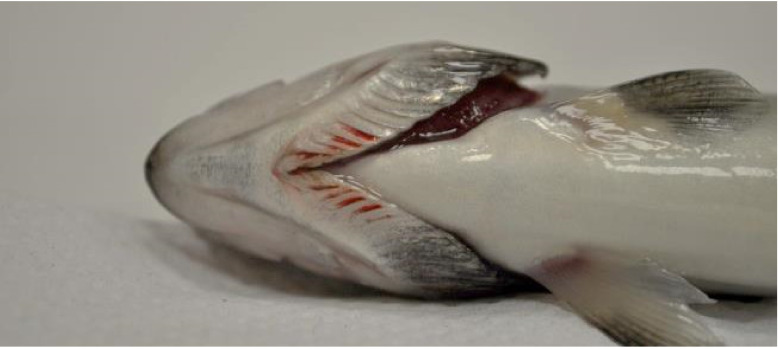
Petechiae visible in the pharyngeal operculum region in the course of ERM.

**Figure 3:**
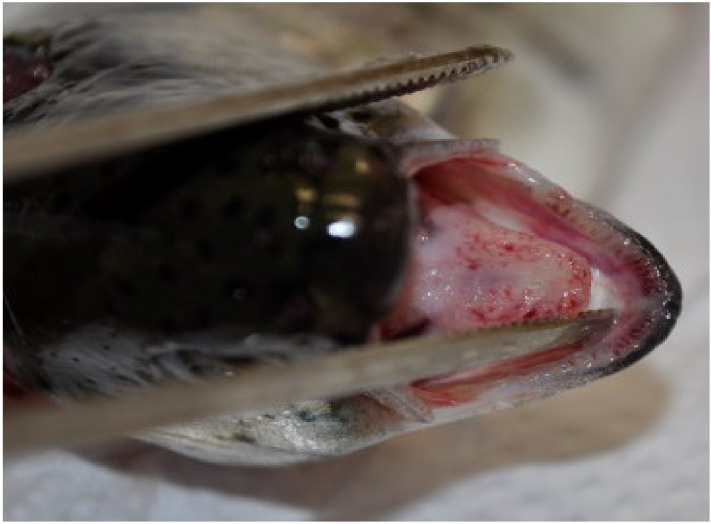
Small petechiae visible in the oral cavity.

**Figure 4:**
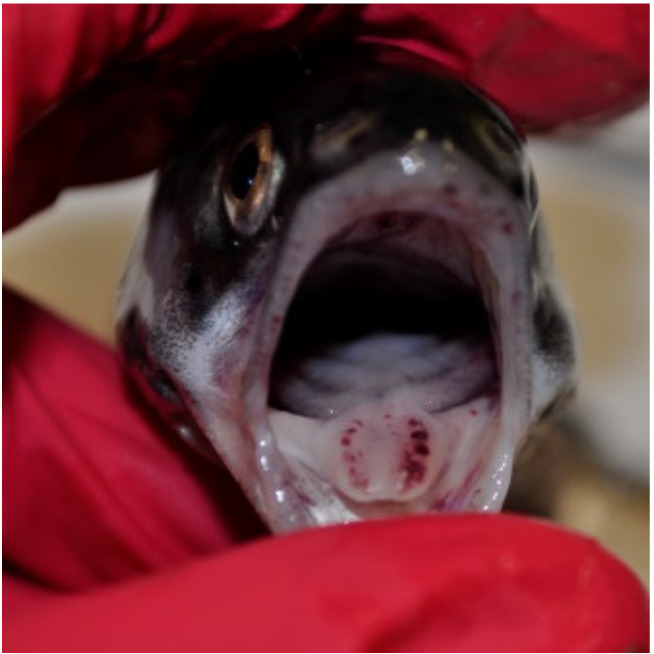
Petechiae visible on the tongue.

Mortality in fish infected with *Y. ruckeri* started three days p.i. and lasted for two weeks. Cumulative mortality in the Yer and Yer+IPN groups reached 47% and 51%, respectively. Only one fish in the IPN group died. No mortality was recorded in the control group. In the groups experimentally infected with *Y. ruckeri*, the first symptoms were observed 24 h p.i., during the first sampling. The fish’s digestive tract was filled with yellow liquid content (Figure 1). In the following days, clinical symptoms characteristic of ERM appeared including petechiae visible in the region of pharyngeal operculum (Figure 2) and inside the oral cavity (Figure 3 and Figure 4).

**Figure 5:**
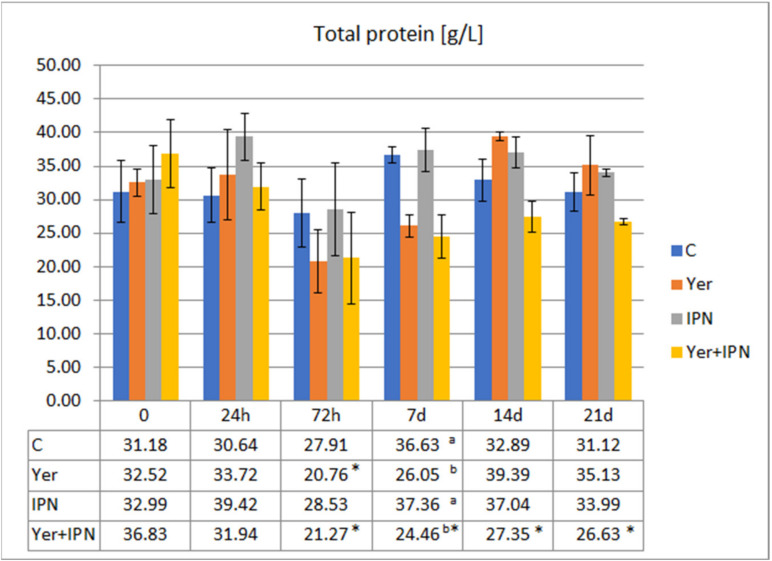
Mean values and standard deviation of total protein levels over the course of the first experimental infection. *—statistically significant differences compared to the day 0 values within a given group (*p* ≤ 0.05); ^a, b^—statistically significant differences between the groups on a given sampling day (*p* ≤ 0.05).

After 72 h p.i., in the Yer and Yer+IPN groups, a decrease in the total protein level was found compared to the level before infection. This trend continued in the Yer+IPN group until the end of the experiment. On day 7 p.i., in the group infected with *Y. ruckeri,* the level of protein was significantly lower than in the other groups (Figure 5).

**Figure 6:**
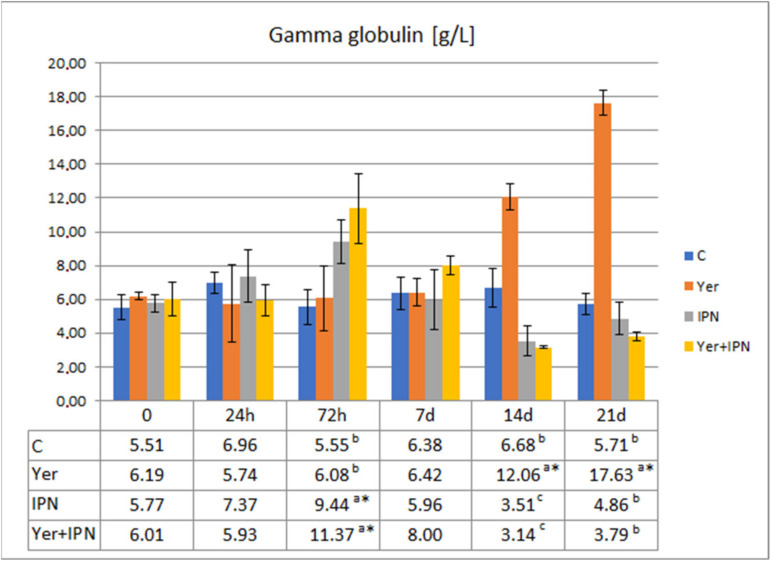
Mean values and standard deviation of gamma globulin levels over the course of the first experimental infection. *—statistically significant differences compared to the day 0 values within a given group (*p* ≤ 0.05); ^a, b, c^—statistically significant differences between the groups on a given sampling day (*p* ≤ 0.05).

A statistically significantly higher gamma globulin level was found in fish infected with IPNV 72 h p.i. and in the Yer group from day 14 p.i. to the end of the experiment. On day 14 p.i., the levels in the IPN and Yer+IPN groups were statistically lower than in the other groups. The level of gamma globulin in the Yer+IPN group was comparable to the level of the IPN group throughout the experiment and statistically differed from the level in the Yer group at three measurement points (Figure 6).

**Figure 7:**
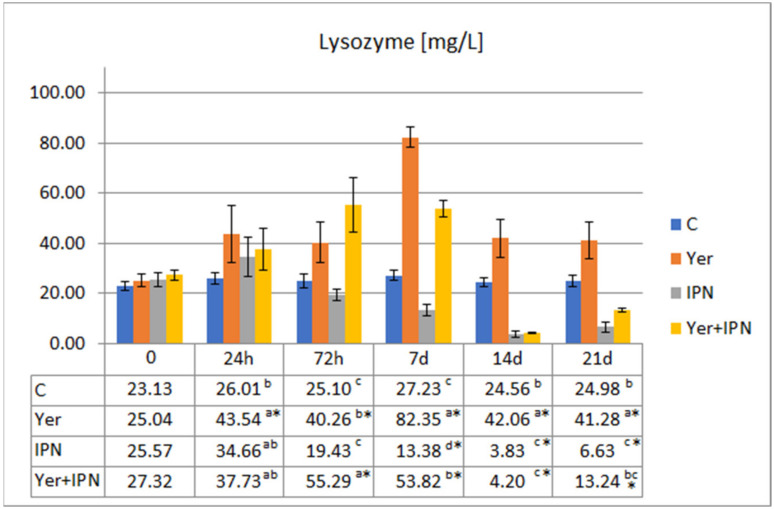
Mean values and standard deviation of lysozyme activity over the course of the first experimental infection. *—statistically significant differences compared to the day 0 values within a given group (*p* ≤ 0.05); ^a, b, c, d^—statistically significant differences between the groups on a given sampling day (*p* ≤ 0.05).

The highest activity of lysozyme was recorded in the Yer and the Yer+IPN groups on day 7 p.i. In the IPN group, a statistically significant decrease in lysozyme activity was noted from day 7 p.i. to the end of the experiment. In the Yer+IPN group, after an initial increase that lasted until day 7 p.i., there was a sharp decrease in the second week (Figure 7).

**Figure 8:**
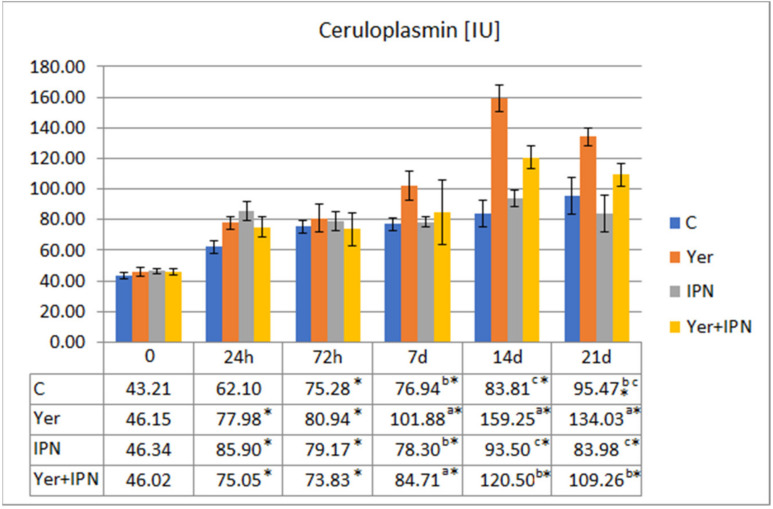
Mean values and standard deviation of ceruloplasmin activity over the course of the first experimental infection. *—statistically significant differences compared to the day 0 values within a given group (*p* ≤ 0.05); ^a, b, c^—statistically significant differences between the groups on a given sampling day (*p* ≤ 0.05).

There was a gradual increase in the ceruloplasmin activity in all groups. On day 7 p.i., the level of activity in the Yer and Yer+IPN groups was significantly higher than in the other groups. In the Yer group, this tendency continued until the end of the experiment. In the Yer+IPN group, the ceruloplasmin activity in the last sampling did not differ statistically from the control, although it was higher than in the group infected with IPNV (Figure 8).

**Figure 9:**
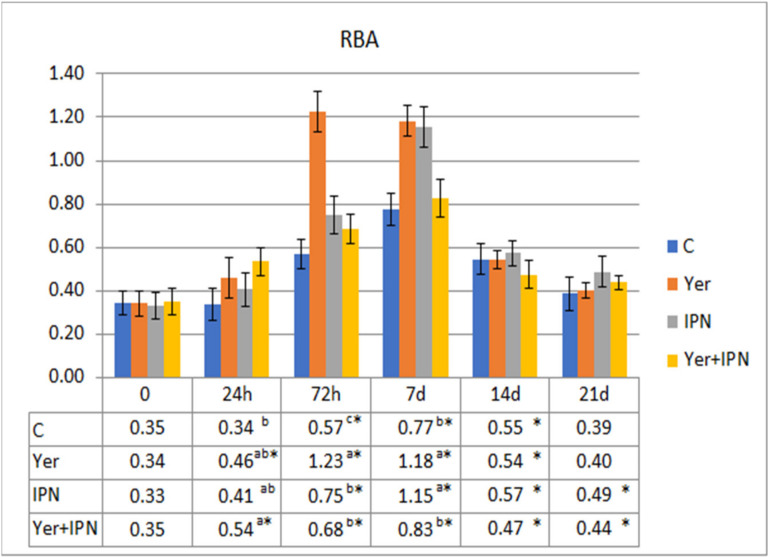
Mean values and standard deviation of metabolic activity of phagocytes over the course of the first experimental infection. *—statistically significant differences compared to the day 0 values within a given group (*p* ≤ 0.05); ^a, b, c^—statistically significant differences between the groups on a given sampling day (*p* ≤ 0.05).

The RBA tended to increase in all groups between 72 h and day 14 p.i. Although the upward trend occurred in all groups, the growth dynamics differed significantly between the groups. The lowest increases were recorded in the control group and the Yer+IPN group (Figure 9).

**Figure 10:**
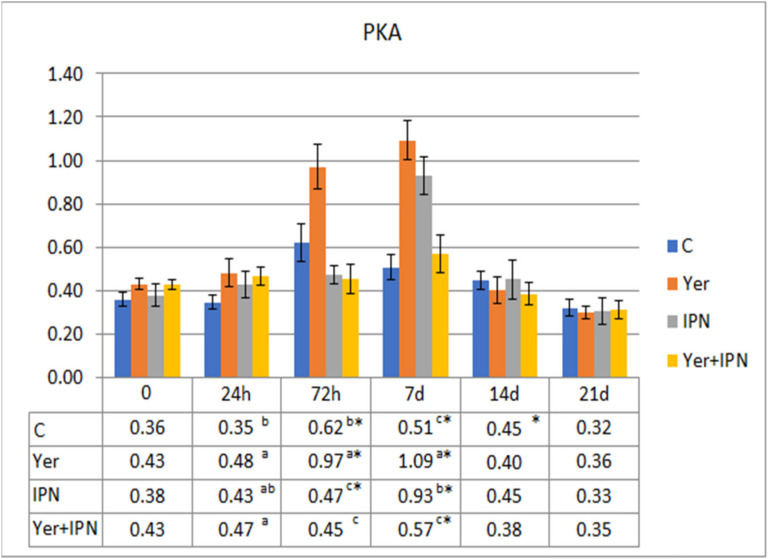
Mean values and standard deviation of potential killing activity of phagocytes over the course of the first experimental infection. *—statistically significant differences compared to the day 0 values within a given group (*p* ≤ 0.05); ^a, b, c^—statistically significant differences between the groups on a given sampling day (*p* ≤ 0.05).

The PKA was highest in the group infected with *Y. ruckeri* from 72 h to day 7 p.i. At 72 h p.i., the response level was significantly lower in both groups infected with the IPNV compared to the control group, although on day 7 the level in the group infected with only the IPNV increased significantly, while the Yer+IPN group did not differ from the control group (Figure 10). 

### 3.2. Experiment II

Two weeks after infection with IPNV, fish were infected with *Y. ruckeri*. Due to the high mortality rate in the groups infected with *Y. ruckeri* (60% in the Yer+IPN group and 62% in the Yer group), samples on day 21 were taken only from the control and IPNV-infected groups. Mortality in the control and IPN groups was 20% and 43%, respectively.

Disease symptoms in the groups infected with *Y. ruckeri* were the same as in the first experiment. In the IPNV-infected groups, deaths were preceded by a short period during which the fish exhibited accelerated gill-lid movement and swimming problems. 

**Figure 11:**
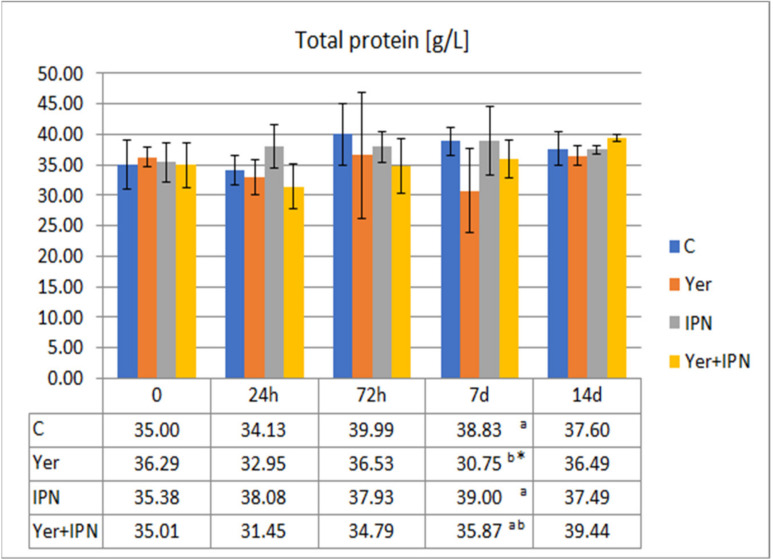
Mean values and standard deviation of total protein levels over the course of the second experimental infection. *—statistically significant differences compared to the day 0 values within a given group (*p* ≤ 0.05); ^a, b^—statistically significant differences between the groups on a given sampling day (*p* ≤ 0.05).

The total protein level of each group is shown in Figure 11. On day 7 p.i., in the Yer group, the total protein level was significantly lower compared to the other groups, as well as to the level before infection.

**Figure 12:**
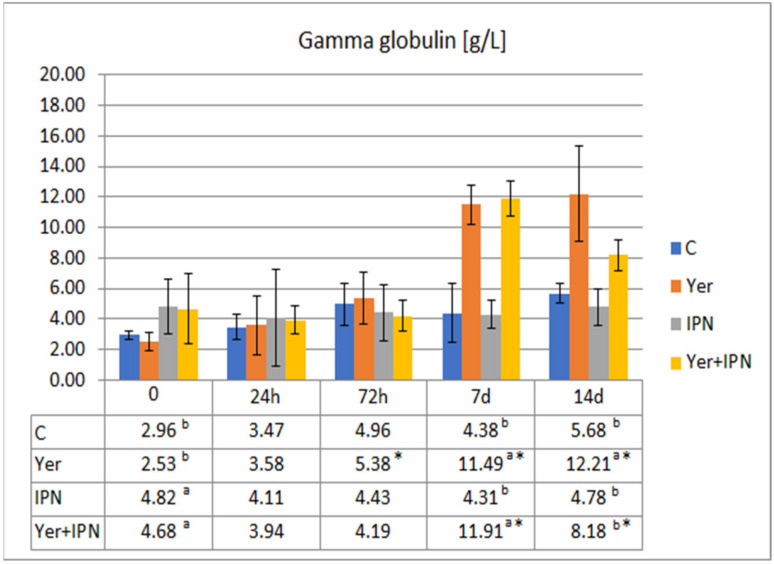
Mean values and standard deviation of gamma globulin levels over the course of the second experimental infection. *—statistically significant differences compared to the day 0 values within a given group (*p* ≤ 0.05); ^a, b^—statistically significant differences between the groups on a given sampling day (*p* ≤ 0.05).

The gamma globulin level was significantly lower in groups previously infected with the IPNV (before *Y. ruckeri* infection). There were no significant differences between the groups in the next two samples. On day 7 p.i. with *Y. ruckeri*, significantly higher levels were recorded in the Yer and Yer+IPN groups. On day 14 p.i., the Yer group showed a significantly higher level of gamma globulin compared to all other groups (Figure 12).

**Figure 13:**
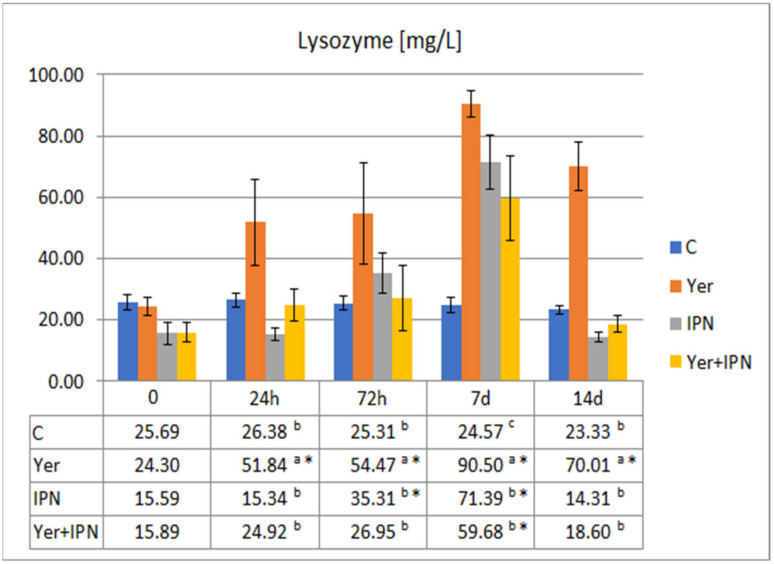
Mean values and standard deviation of lysozyme activity over the course of the second experimental infection. *—statistically significant differences compared to the day 0 values within a given group (*p* ≤ 0.05); ^a, b, c^—statistically significant differences between the groups on a given sampling day (*p* ≤ 0.05).

On the day of *Y. ruckeri* infection, no statistically significant differences in lysozyme activity were found between the groups. The highest activity and the highest growth dynamics were observed in the Yer group. The Yer+IPN and IPN groups did not differ from each other at any time point, and only on day 7 p.i. the activity of lysozyme in these groups was higher than in the control group (Figure 13).

**Figure 14:**
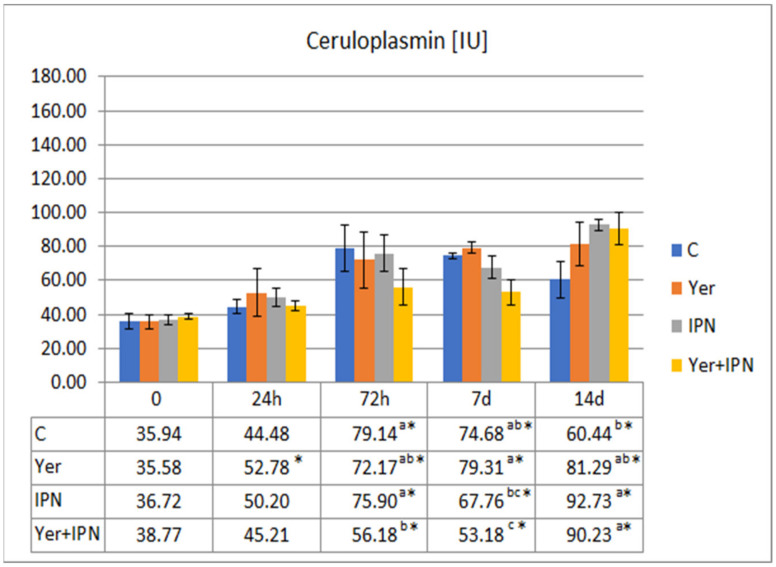
Mean values and standard deviation of ceruloplasmin activity over the course of the second experimental infection. *—statistically significant differences compared to the day 0 values within a given group (*p* ≤ 0.05); ^a, b, c^—statistically significant differences between the groups on a given sampling day (*p* ≤ 0.05).

Ceruloplasmin activity showed an upward trend in all experimental groups. In the Yer+IPN group, the response was lower 72 h p.i. compared to groups C and IPN and on day 7 in reference to groups C and Yer. On day 14, a significantly higher level of ceruloplasmin activity as compared to the control was noted in the Yer+IPN and IPN groups (Figure 14).

**Figure 15:**
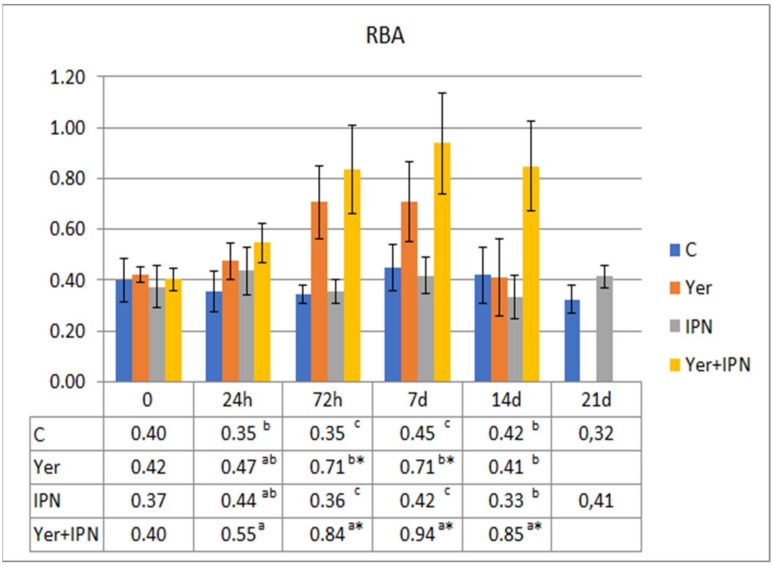
Mean values and standard deviation of metabolic activity of phagocytes over the course of the second experimental infection. *—statistically significant differences compared to the day 0 values within a given group (*p* ≤ 0.05); ^a, b, c^—statistically significant differences between the groups on a given sampling day (*p* ≤ 0.05).

**Figure 16:**
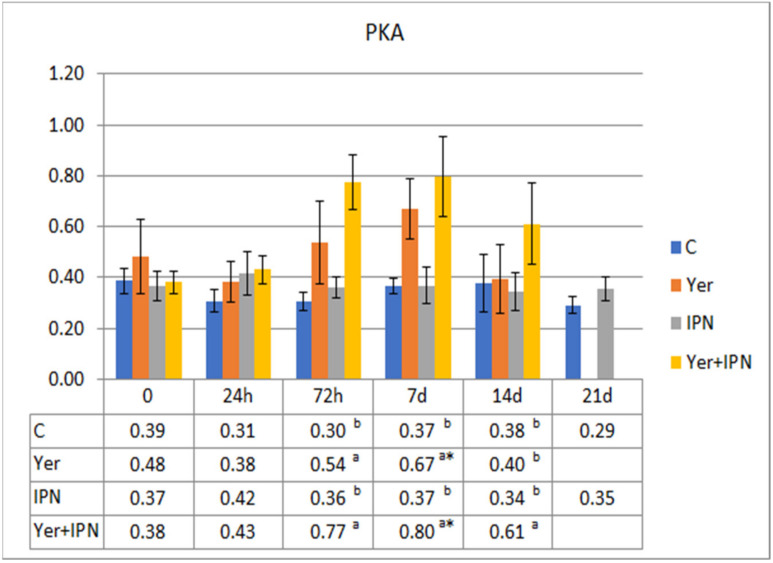
Mean values and standard deviation of phagocyte-killing activity over the course of the second experimental infection. *—statistically significant differences compared to the day 0 values within a given group (*p* ≤ 0.05); ^a, b^—statistically significant differences between the groups on a given sampling day (*p* ≤ 0.05).

The metabolic activity of phagocytes in the control and IPN groups did not change significantly with time. There were also no differences between these groups at any time point. An upward trend was shown in the Yer+IPN group and Yer group from 72 h to 7 days p.i. Phagocyte activity in the Yer+IPN group was significantly higher compared to all other groups from 72 h to 14 days p.i. (Figure 15). Similar observations were found in the potential killing activity of phagocytes (Figure 16).

## 4. Discussion

Many experiments using animal models have tested the relationship between bacteria and viruses over the course of infection. To cause disease, microorganisms must cross the mucosal barrier, penetrate the tissues, multiply in the tissues, inhibit the host’s defenses, and damage the host’s cells. Viruses can assist pathogenic bacteria in one or more of the above-mentioned steps. Disease exacerbation, greater severity of tissue damage, or greater mortality have been observed in animals exposed to bacterial contamination in combination with a viral infection. In the case of fish diseases, studies that would determine the correlation between infection with viral and bacterial pathogens are limited, and there are no studies that would determine whether IPNV infection affects the pathogenicity of *Y. ruckeri*.

The conducted experiments were aimed at comparing the course of *Y. ruckeri* and IPNV co-infections to infections caused by a single pathogen. In the first experiment, infection with both pathogens was performed on the same day. Under natural conditions, such a situation may take place when the fish are transported to another breeding facility where these pathogens persist endemically. In the second experiment, the infection with the IPNV was carried out two weeks before the infection with *Y. ruckeri*, which corresponds to a situation where a transported IPNV carrier fish would become infected with *Y. ruckeri* at the farm. Intraperitoneal injection was chosen as the method of infection. The advantage of this method is having the same infectious dose for all fish and greater infection efficiency. However, this route of infection bypasses important defense barriers (both specific and non-specific) associated with skin and mucus [21,22]. The injection itself is also not indifferent to the body, which is reflected in the increased activity of ceruloplasmin in the control group.

Studies on co-infection with IPNV and *Vibrio* (*V.*) *carchariae* in the grouper (*Epinephelus sp.*) indicated a synergistic effect of these pathogens and the occurrence of mass deaths [17]. A similar trend was demonstrated in Atlantic salmon over the course of *V. salmonicida* infection in fish during the acute phase of IPNV infection [23]. The current results do not confirm this relationship in the case of IPNV and *Y. ruckeri* infection. Mortality in the Yer+IPN group in both experiments did not differ significantly from that in the group infected only with the bacterial pathogen. Higher mortality rates were noted in the second experiment in all groups, including control fish. This may be due to the fact that the overall condition of the fish in the second experiment was lower than in the first experiment that was carried out earlier. This creates some difficulties in comparing groups between experiments, so in discussing the results, the main focus will be on comparing trends between groups in a given experiment.

The level of total protein in the first experiment ranged from 20.76 to 39.39 g/L, while in the second it ranged from 30.75 to 39.99 g/L. These ranges are within the reference values (20–60 g/L) given by Wedemeyer and Chatteron [24]. Other authors report ranges of 36–56 g/L, 40–68 g/L, and 31–49 g/L [25,26,27]. There were no statistically significant changes in the control group or the IPNV infected group, neither in the first nor in the second experiment. In the group infected with *Y. ruckeri*, hypoproteinemia was noted 72 h after infection in the first experiment and on day 7 of the second experiment. The Yer+IPN group in the first experiment showed a reduced level of total protein from 72 h after infection, persisting until the end of the experiment. In the second experiment, the level of protein did not differ significantly over time within the group or from other groups. Decreased protein levels may be due to increased protein catabolism in the acute phase of infection or decreased protein production due to liver damage. Hypoproteinemia over the course of ERM has also been reported by other authors [28,29]. With the simultaneous infection of both pathogens, the reduced protein level was maintained longer than in the group infected only with *Y. ruckeri*, which may indicate a synergistic effect of the studied pathogens.

There were significant differences in the gamma globulin level. Over the course of the first experiment, the Yer+IPN group did not differ significantly from the IPN group. Both these groups showed an increase in the level of gamma globulin 72 h after infection, however, later it decreased to a level comparable to the control group. In both experiments, an upward trend of gamma globulin level was observed in the Yer group, which was most visible from day 14 after infection in the first experiment and from day 7 in the second experiment. In the second experiment, the Yer+IPN group on day 7 did not differ from the group infected with *Y. ruckeri*. The obtained results may indicate that the development of specific immunity against ERM in the case of co-infection depends on the time elapsed between infections. The development of immunity may be inhibited when infections occur in a short time, but long-term IPNV carrier state does not have a major impact on immunity against ERM. 

Similar results were obtained by Bruno and Munro [30], who showed no effect of IPNV infection on post-vaccine immunity. Infection with IPNV preceded vaccination by about two months, and infection with *Y. ruckeri* was performed after another two months. Studies on Atlantic salmon also showed no effect of being an IPNV carrier on post-vaccine immunity against vibriosis or furunculosis [31].

Lysozyme activity is one of the key components of non-specific immunity. Its level depends on many factors, such as sex, age, and size, water temperature, season of the year, pH, presence of toxic substances, level of stress, and infection with various pathogens. The stress response depends on the duration and type of stress factor. Both an increase and a decrease in the level of lysozyme activity induced by transport and breeding treatments in rainbow trout have been reported [32]. Due to the multitude of factors affecting lysozyme activity, it is difficult to define reference values for this indicator. Over the course of bacterial infection, there is usually an increase in lysozyme activity, which was noted, among others, over the course of infection with *A. punctata*, *A. salmonicida*, or *E. tarda* [33,34,35]. The obtained results showed that over the course of *Y. ruckeri* infection a significant increase in lysozyme activity also occurred and reached the highest level 7 days p.i. and then decreased. 

In the case of the Yer+IPN group, in both the first and the second experiment, an increase in lysozyme activity was noted, however, the response was significantly lower than in the group infected with only *Y. ruckeri*, and a sharp decrease was observed on day 14 p.i. Over the course of the first experiment, a strong suppressive effect of the IPNV on lysozyme activity was demonstrated. In the second experiment, there was an increase on day 7 p.i. and a decrease similar to the Yer+IPN group and found in the next sampling. Infection with the IPNV has been shown to reduce lysozyme activity, which has a bactericidal effect. This may indicate a synergistic effect of the pathogens. 

There was an increase in ceruloplasmin activity in all groups, but the dynamics of this increase differed both between groups and between experiments. In the first experiment, ceruloplasmin activity increased significantly in all groups, except for the control group, 24 h after infection. In the control group, an increase was observed in subsequent sampling. There are relatively little data on the level of ceruloplasmin activity in bacterial fish diseases, but an increase in ceruloplasmin gene expression in the liver of channel catfish *(Ictlurus punctatus*) infected with *E. ictaluri* in 24 h and rohu (*Labeo rohita*) infected with *A. hydrophila* in six hours was noted [36,37]. The results obtained in the current research indicate that an increase in the activity of this indicator was also observed in rainbow trout over the course of ERM. 

There were no statistically significant differences between the control and the group infected with IPNV in the first experiment. In the second experiment, as in the case of lysozyme activity, the group previously infected with IPNV reacted to the simulation of infection more strongly than the control group. High variability in the response to *Y. ruckeri* infection was demonstrated. In the first experiment, the Yer group had the highest level of ceruloplasmin activity, while it did not differ statistically from the control group in the second experiment. This result is difficult to interpret and may be connected to differences in the overall condition of fish. 

The phagocytic activity of macrophages is involved in the body’s defense against both bacterial and viral infections [38,39]. Similar trends were observed for both the metabolic and killing activity of phagocytes. In the first experiment, these parameters increased significantly in all groups. With the simultaneous infection of *Y. ruckeri* and IPNV, the phagocyte response was suppressed, which was particularly evident on day 7 p.i. In the case of an earlier infection with IPNV, the situation was the opposite and in the Yer+IPN group, the phagocyte response was the strongest, which indicates a significant role of the time elapsed between infections in the development and course of co-infections. Studies carried out on isolated macrophages of Atlantic salmon indicated a possible suppressive effect of IPNV and a reduction in the activity of the respiratory burst [40]. Although the suppressive effect of the IPNV on phagocytic activity in a single infection was not confirmed, it occurred in the case of co-infection with *Y. ruckeri* in the first experiment.

Infection with the IPNV is widespread in salmonid farming around the world. It is related to, inter alia, a carrier state that develops after disease and may persist for years. The virus can avoid the immune system and persist inside leukocytes for a long time [38]. Often its titer is so low that it is not detected with standard diagnostic methods [31,41,42]. 

The mechanisms of interaction between pathogens in vivo within the host organism are complex and relatively poorly understood. There are many reports of the antagonistic effect of IPNV on the development of infection with other viruses, which is referred to as “superinfection”. Primary IPNV infection has been shown to protect fish against viruses such as the viral hemorrhagic septicemia virus, infectious hematopoietic necrosis virus, and infectious salmon anemia virus (ISAV)). This may be due to the stimulation of interferon production by the IPNV, which protects against the development of infection with other viruses, or a faster replication rate of this virus [15,38,43]. 

In the co-infection of the IPNV with bacterial agents, it is assigned a synergistic role due to the induced immunosuppression. However, the obtained results indicate that the role of the IPNV in co-infection depends on the time elapsed between infections, and its suppressive effect is limited to few weeks after infection. This confirms the results of studies conducted on Atlantic salmon, where the influence of IPNV infection on ISA and *V. salmonicida* infection was studied. In a situation where IPNV infection preceded the next infection by three weeks, lower mortality due to ISAV infection and higher mortality in mixed infection with *V. salmonicida* was observed. However, when the time between infections was extended to six weeks, no statistically significant differences were found [23].

## 5. Conclusions

Although it is difficult to determine the influence of IPNV infection on the pathogenicity of *Y. ruckeri*, decreased parameters of the non-specific response, such as phagocyte killing and metabolic activity and lysozyme activity over the course of mixed infection in the first experiment, may indicate a synergistic effect in the case of infection with both agents at a similar time.

## Data Availability

All the data discussed in the manuscript are present within the text and tables.
